# Effects of fragment traits, burial orientation and nutrient supply on survival and growth in *Populus deltoides* × *P. simonii*

**DOI:** 10.1038/srep21031

**Published:** 2016-02-15

**Authors:** Ping Zhang, Zhi-Qin Su, Lie Xu, Xue-Ping Shi, Ke-Bing Du, Bo Zheng, Yong-Jian Wang

**Affiliations:** 1 College of Horticulture & Forestry Sciences, Huazhong Agricultural University, Wuhan 430070, China

## Abstract

Clonal propagations of shoot or root fragments play pivotal roles in adaptation of clonal trees to environmental heterogeneity, i.e. soil nutrient heterogeneity and burials after disturbance. However, little is known about whether burial orientation and nutrient supply can alter the effects of fragment traits in *Populus*. Shoot and root fragments of *Populus deltoides* × *P. simonii* were subjected to burials in two different fragment diameters (0.5 and 2.0 cm), two fragment lengths (5 and 15 cm) and three burial orientations (horizontal, upward and downward). For the shoot fragments, survival and growth were significantly higher in the larger pieces (either in length or diameter) and the horizontal/upward burial position. On the contrary, the effect of burial position was reversed for the root fragments. Shoot/root fragments of 15 cm in length in horizontal burial position were then subjected to two different fragment diameters (0.5 and 2.0 cm) and four types of nutrient supplies (without nutrient, low frequency, high frequency and patchy). Growth of shoot fragments of 2.0 cm in diameter significantly increased in high frequency and patchy nutrient supplies than that of without nutrient treatment. These results suggest that burial orientation and nutrient supply could be employed in clonal propagations of cuttings, afforestation or regeneration in *Populus*.

The capability of clonal propagations in small fragments to regenerate and grow is considered to be important for successful establishment in clonal plants^1^,^2^. Some clonal trees showed clonal propagation by producing new ramets along rhizomes or roots^3^,^4^,^5^,^6^, or by forming adventitious root from shoot or root of fragments (i.e. sprouting and root suckering)^6^,^7^,^8^, to allow plants to adapt to adverse conditions, such as nutrient or water shortage and disturbance^3^,^9^,^10^. In poplar species, shoot or root cuttings is a simple and efficient way for vegetation re-establishment. The main traits influencing the propagating potential of fragments are the size (length and diameter) of the shoots or roots^11^,^12^, because reserves (e.g., nonstructural carbohydrates and soluble proteins) stored in shoot or root fragments that being an important short-term storage organ, can be remobilized and reused for the recovery^13^,^14^. Increasing fragment length or diameter may facilitate the survival and growth of clonal fragments because internode length and diameter may be positively correlated with the amount of reserves stored^14^,^15^,^16^. These reserves need to last through the development of the emerging sprout, until it is ready for photosynthesis. Thus, very thin root cuttings may lack sufficient nutritional reserves or the ability to regenerate shoot primordial^11^,^17^,^18^.

Disturbance such as deforestation (clear-cutting), fire and landslips can disrupt plant structures and modify environmental conditions, such as soil nutrients and burial orientation or depth^19^. Following these forest disturbances, suckers produced from the residual shoot or root system of the previous stand form a new forest stand^2^,^10^,^20^,^21^. Thus, burial orientation in soils may greatly affect survival and growth of clonal fragments because both biotic (e.g., pathogen activities) and abiotic conditions (e.g., nutrient, temperature and moisture) were changed in buried parts of fragments^22^,^23^. Moreover, after deforestation, such small clonal fragments might become positioned in orientations away from the horizontal, which is the typical orientation for re-growth for Poplar. Departure from the horizontal position might induce asymmetric distribution of endogenous growth substances, such as auxins, in the new buds of shoot or root fragments, and make them reorient toward the vertical owing to gravity stimulation^16^,^23^. This could increase reliance on stored resources in fragments. In addition, the horizontal might also increase the forming of adventitious roots due to the facilitation of the biotic and abiotic conditions in soil to both the proximal and distal parts of fragments.

Simultaneously, spatial heterogeneity in soil nutrients is common in natural habitats^24^,^25^, especially in disturbed environment. Shoot or root fragments of clonal trees frequently root in microsites with contrasting levels of nutrients, and one common effect of nutrient heterogeneity is to increase plant performance after rooting^26^. This may be due at least partly to greater efficiency of uptake at higher concentrations of nutrients. Patchiness of soil nutrients can also influence the survival and growth of horizontal clonal fragments^14^,^16^,^25^. Because clonal plants can concentrate their roots where nutrients are high, and allow the exchange of resources including carbohydrates, water and nutrients to function as a physiologically integrated system^3^,^4^,^27^,^28^,^29^. This physiological integration has been revealed to strengthen the behavior of fragments suffering from different local stresses such as nutrient depletion^30^,^31^, burial^3^,^27^, and soil erosion^3^,^4^. Nutrient heterogeneity could also act as a trigger for forest regeneration after disturbance^1^,^32^,^33^,^34^. Nitrate is known to facilitate the biosynthesis of cytokinins^32^,^35^,^36^, which are known to be involved in sucker initiation in aspen^1^,^32^.

As a result of global climate change, especially for the variation in precipitation and temperature, the variation of water and nutrients availability is expected to increase in spatio-temporal scale^37^,^38^. These heterogeneous changes could have significant direct effects on survival and growth of fragment propagation in natural re-establishment and afforestaion in Poplar.

In this study, we discussed whether fragment traits, burial orientation and nutrient supply will affect emergence ability, survival and growth in Poplar. Therefore, we put shoot and root fragments with different diameters and lengths of a clonal Poplar tree (*P. deltoides* × *P. simonii*) under different burial orientations and nutrient supplies to analyze the effects on the survival and growth, respectively. In the first experiment, we subjected two types of fragments (shoot vs. root) to twelve treatments differing in fragment diameter (0.5 vs. 2.0 cm), fragment length (5 vs. 15 cm) and burial orientation (horizontal vs. upwards vs. downwards). In the second experiment, we set 15 cm in length shoot or root fragments in horizontal burial position with different fragment diameters (0.5 vs. 2.0 cm) and nutrient supplies (without nutrient (CK) vs. low frequency vs. high frequency vs. patchy). The survival and growth of fragment with different traits might vary depending on burial orientation and nutrient supply. Therefore, we tested our three hypotheses: (1) Fragment traits will affect survival and growth of *P. deltoides* × *P. simonii*, and survival and growth may be improved greater in shoot fragments with larger diameter and/or longer length; (2) burial orientation will alter the interactive effects of fragment type (shoot vs. root), fragment diameter and fragment length on survival and growth, i.e. survival and growth of the shoot fragment with larger diameter and longer length may be improved greater in the horizontal and upward burial position than that in the downward position; (3) nutrient supply will change the interactions of fragment type and fragment diameter on survival and growth, i.e. high frequency and patchy nutrient supplies may result in higher survival and growth rate in the shoot fragment with larger diameter.

## Results

### Effects of fragment type, fragment diameter, fragment length and burial orientation

**Figure 1:**
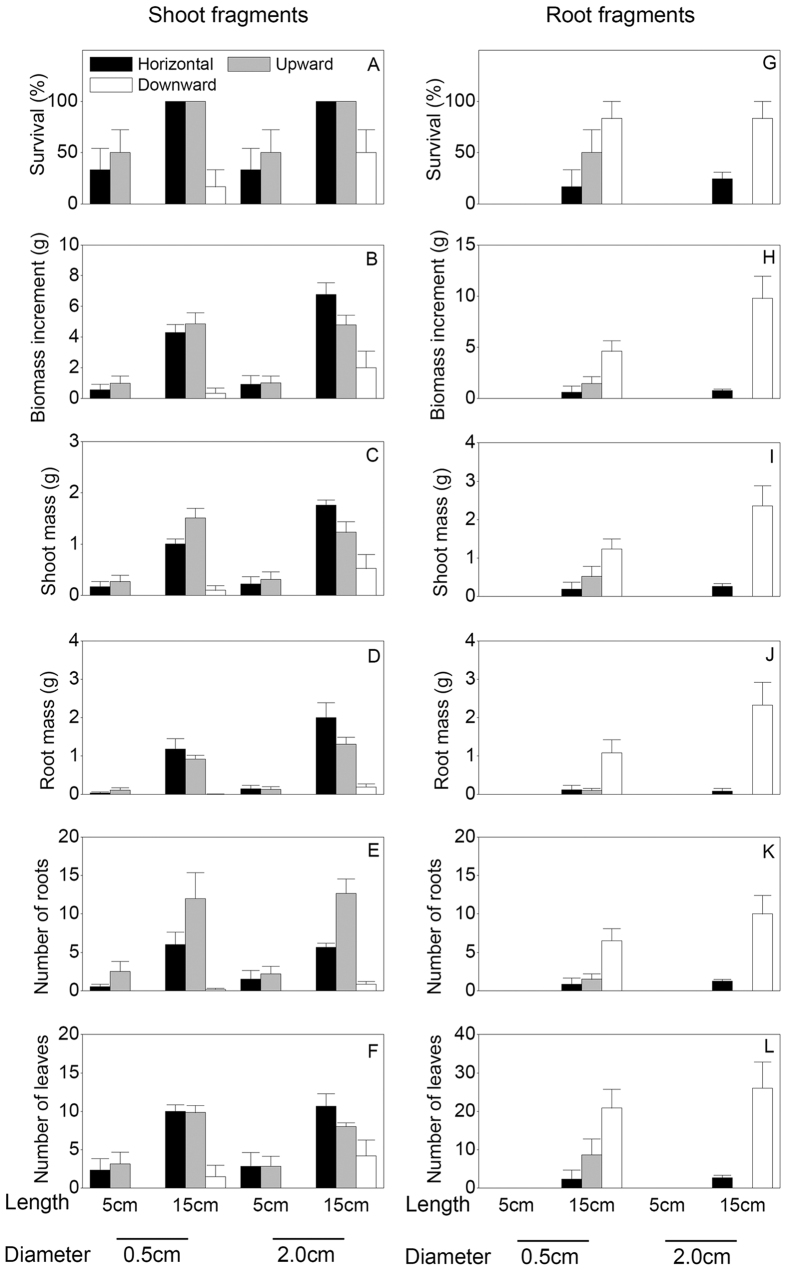
Effects of fragment type, fragment diameter, fragment length and burial orientation on survival (**A,G**), biomass increment (**B,H**), shoot mass (**C,I**), root mass (**D,J**), number of roots (**E,K**) and number of leaves (**F,L**) of *P. deltoides* × *P. simonii* at harvest. Means +1 SE are given.

Fragment type and fragment length significantly affected the survival and growth measures of *P. deltoides* × *P. simonii* (Table 1). Survival, biomass increment, shoot mass, root mass and number of roots were significantly greater in the shoot fragment treatments than those in the root fragment treatments (*P* < 0.05), and greater in the 15 cm in length fragment treatments than those in the 5 cm in length fragment treatments (*P* < 0.05) (Table 1, Fig. 1).

Fragment diameter significantly influenced biomass increment, shoot mass and root mass of *P. deltoides* × *P. simonii* (Table 1). Values of these three measures were significantly higher in the 2.0 cm in diameter treatments than those in the 0.5 cm in diameter ones (Fig. 1). Thus 2.0 cm in diameter & 15 cm in length fragments accumulated the greatest biomass increment, shoot mass and root mass, especially in horizontal burial conditions (Fig. 1B–D).

Burial orientation alone had no significant effects on any of measures of *P. deltoides* × *P. simonii* (Table 1), instead the effects of burial orientation on all measures significantly depended on fragment type (Table 1, *P* < 0.05 for Fragment type (T) × Burial orientation (O)) and on fragment type and fragment length (Table 1, *P* < 0.05 for T × Fragment length (L) × O). Of the shoot fragments, horizontal and upward burial positions improved the survival and growth greater than downward burial position (*P* < 0.05) (Table 1; Fig. 1A–F); of the root fragments, downward burial position significantly increased the measures than in horizontal or upward position in 15 cm in length treatments (*P* < 0.05), but not for the 5 cm in length ones (*P* > 0.05) (Table 1, Fig. 1G–L). Simultaneously, treatments of 15 cm in length shoot fragment in horizontal and upward positions greatly increased the survival and growth measures compared to those of 5 cm in length shoot fragments (*P* < 0.05), but not for shoot fragments in downward position (*P* > 0.05) (Table 1; Fig. 1A–F). On the contrary, treatments of 15 cm in length root fragments had significantly higher survival and growth measures than those 5 cm in length root fragments in downward burial position (*P* < 0.05), but not for fragments in horizontal or upward positions (*P* > 0.05) (Table 1, Fig. 1G–L). These results suggest that burial orientation altered the effects of fragment type and fragment length of *P. deltoides* × *P. simonii*.

Moreover, there were interactive effects of fragment diameter (D) × L, D × O, L × O and D × L × O on biomass increment and shoot mass of *P. deltoides* × *P. simonii* (Table 1). Irrespective of fragment type, fragments of 2.0 cm in diameter and 15 cm in length significantly increased biomass increment and shoot mass in horizontal burial position compared to upward position (*P* < 0.05), but the differences were not significant between fragments of 0.5 cm in diameter and 15 cm in length (*P* > 0.05) (Fig. 1B,C,H and I). Biomass increment and shoot mass in the 5 cm in length fragments were not significantly between horizontal and upward burial positions regardless of the diameter (0.5 or 2.0 cm) (*P* > 0.05; Fig. 1B,C,H,I). The results indicate that burial orientation partly altered the effects of fragment length and fragment diameter of *P. deltoides* × *P. simonii*.

### Effects of fragment type, fragment diameter and nutrient supply

**Figure 2:**
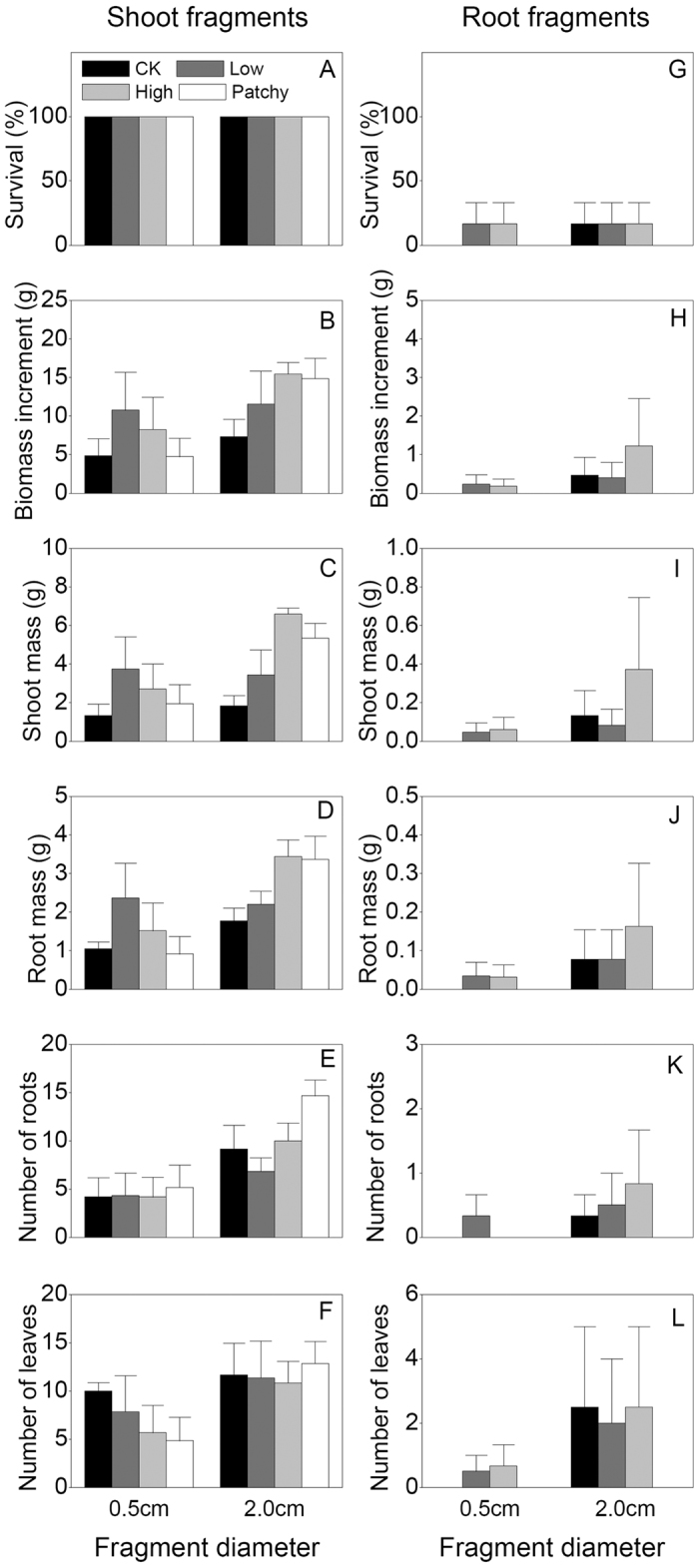
Effects of fragment type, fragment diameter and nutrient supply on survival (**A,G**), biomass increment (**B,H**), shoot mass (**C,I**), root mass (**D,J**), number of roots (**E,K**) and number of leaves (**F,L**) of *P. deltoides* × *P. simonii* at harvest. Means +1 SE are given.

Fragment type, fragment diameter and their interactions (T × D) significantly influenced all measures of *P. deltoides* × *P. simonii* except for effect of fragment diameter and the interactions on the survival (Table 2). Survival and growth were also significantly greater in the shoot fragments than those in the root fragments (*P* < 0.05) (Table 2, Fig. 2). Growth of the 2.0 cm in diameter shoot fragments were significantly greater than the 0.5 cm in diameter ones, but there was no significant difference between the root fragments of 0.5 and 2.0 cm in diameter (Table 2, Fig. 2).

Moreover, effects of nutrient supply on shoot mass and root mass significantly depended on fragment type (*P* < 0.05 for T × Nutrient supply (N), Table 2). Nutrient supply treatments showed that high frequency and patchy nutrient supplies greatly improved the growth of the shoot fragments of 2.0 cm in diameter, compared to without nutrient supply (CK) (*P* < 0.05), but no significant differences were observed in the shoot fragments of 0.5 cm in diameter (*P* > 0.05, Table 2; Fig. 2A–F). However, in the root fragments, all measures were not significantly different between the nutrient supply treatments regardless of the diameters (*P* > 0.05; Fig. 2G–L). Simultaneously, markedly biomass increase of the shoot fragments of 2.0 cm in diameter was associated with increasing nutrient (Fig. 2). These results suggest that nutrient supply partly altered the effects of fragment type and fragment diameter of *P. deltoides* × *P. simonii* in horizontal burial position.

## Discussion

Larger and longer shoot fragments improved survival (100%) and growth (i.e. producing higher biomass increment, mass and number of shoot and root) of *P. deltoides* × *P. simonii* in different burial orientation and nutrient supply treatments. These results suggest that fragment traits are of significant importance for this species in heterogeneous environments. Also, horizontal and upward burial orientations improved the survival and growth of shoot fragments greater than downward burial orientation, especially the shoot fragments of 15 cm in length, while the situation was reversed in the root fragments of 15 cm in length. Moreover, larger fragments, 2.0 cm in diameter and/or 15 cm in length, significantly increased biomass increment and shoot mass in the horizontal burial orientation compared to upward one, especially for the shoot fragments. Thus, the results partly support our first two hypotheses, suggesting that shoot fragments with larger diameter and longer length will improve the survival and growth, and burial orientation will change the interactive effects of fragment type, fragment diameter and fragment length on *P. deltoides* × *P. simonii*.

Clonal propagation of Poplar fragments depends on the ability of adventitious root development. The adventitious roots are mainly developed from stem or root fragments that have been injured or stimulated by hormones or pathogenic microorganisms^39^. Previous studies have indicated that fragments with larger attached shoot had higher activity in promoting adventitious root development and survival rate, thus achieved faster growth both in many *Populus* species^11^,^12^,^39^,^40^ and in other clonal trees^17^,^18^. This was consistent with the hypothesis that larger fragments (cuttings) will accumulate more storages (nutrients, carbohydrate and hormonal content) to stimulate plant rooting and growth^16^,^17^,^18^,^39^. Commonly, the diameter or position could also strongly influence the rooting ability of fragments. Fragments with large diameter (base of the shoot) may have higher potential to form adventitious roots than that from tops^11^,^12^,^39^. Moreover, rooting and survival of fragments undergo complex regulation by multiple hormones, i.e. auxin and cytokinins, depending on fragment length and diameter. And lots of evidence supports the role of plant growth regulators in root development^13^,^23^. Therefore, fragments type, diameter (position from donor tree) and length play important roles in rooting process and growth of poplar hardwood fragments.

However, fragments diameter and length did not influence the survival and growth performance of root fragments. *P. deltoides* × *P. simonii* did not root well from root fragments, unlike in aspen spieces *P. tremuloides* and *P. tremula*^11^,^12^. There are several features, such as hormones levels (e.g., ratio of auxins to cytokinins), carbohydrates availability and physical condition, may be more important in successful propagation from root fragments^12^,^40^.

Burial orientation played a significant role in propagation of shoot and root fragments in *P. deltoides* × *P. simonii,* especially shoot fragments with larger diameter and longer length. Survival of shoot fragments in size of 15 cm in length and 2.0 cm in diameter were greatest among all treatments when ramets were oriented in the horizontal position. The results indicated that horizontal positioning can increase the establishment of long and thick clonal fragments, which might be created and dispersed by environmental or artificial disturbance, or by fragment propagation in afforestation. Our results may provide the first published suggestion that tendency to lodge in the horizontal position could be advantageous in *Populus*. One possible explanation is that shoot fragments in horizontal burial position actually have better storage utilization and greater ability to respond to favorable conditions^15^,^16^. Also, being positioned downward can improve the establishment of root fragments (83% survival), indicating that positive effects of this treatment on survival are slightly higher rooting efficiency in the distal part and better storage of reserves^40^. The survival for root fragments under downward burial in our experiments are similar to some published studies in Poplar^11^,^12^.

In the second half of our experiment, high frequency and patchy nutrient supplies to the shoot fragments of 2.0 cm in diameter greatly improved biomass measures and number of roots. Moreover, the amount and frequency of nutrient supply were positive related to three biomass measures. Thus, our results partly support the third hypothesis, suggesting that high-frequency and patchy nutrient supplies promote growth of the shoot fragment in larger diameter in *P. deltoides* × *P. simonii.*

Although high levels of nutrient did not contribute to the establishment of rootings, high soil fertility and minerals can thereafter increase root growth, morphology and architecture^19^,^39^. Nitrogen forms (such as nitrate) could stimulate the biosynthesis of cytokinins^1^,^35^,^36^, which might promote development of fine-root and growth of shoot and root fragments in Poplar forest^1^,^32^. Furthermore, fragment growth after rooting may be more dependent on environmental conditions, such as nutrition and water^18^.

Simultaneously, many studies generally indicate positive influences of spatial heterogeneity in nutrient supply on the growth of clonal fragments^25^,^41^. In patchy nutrient, fragments accumulated greater biomass than those in homogeneous nutrient^25^. The underlying mechanism might result from basipetal transport of nutrients (from distal part of the shoot fragments to proximal part) in shoot of *P. deltoides* × *P. simonii* to improve rooting and root growth. That also allows fragments to efficiently uptake resources and such resources are re-distributed within the fragments through physiological integration to increase the survival and growth of the whole fragment^41^. Physiological integration and resource transportation have been shown to enhance the performance of fragments suffering from different local stresses (i.e. nutrient shortage and burial^3^,^27^,^31^). Moreover, physiological integration and resource transportation allowed the exchange of resources such as carbohydrates, water and nutrients^3^,^4^,^27^,^28^,^29^. The results further supported the source-sink hypothesis, suggesting that differences in nutrient supply drive the sharing process, with resources moving from the part with high resources availability to those with low resources availability.

Global change may lead to resource heterogeneity, such as variation of nutrients and water^37^, which may have significant direct or indirect effects on survival and regeneration of fragment propagation after disturbance. Therefore, multiple modules from both heterogeneous environment and internal fragment traits play a pivotal role in survival and growth in *P. deltoides* × *P. simonii*. The treatment of the shoot fragments (15 cm in length, and 2.0 cm in diameter) in upward/horizontal burial position might be advantageous and used in clonal propagation in *P. deltoides* × *P. simonii*. To sum up, larger size of shoot fragment under upward or horizontal burial positions and under high or heterogeneous nutrient supplies will favor root development and growth in *P. deltoides* × *P. simonii*, which could be an alternative method in clonal propagation of cuttings, afforestation or regeneration in poplar.

## Materials and Methods

### Ethics statement

The experimental plants were obtained from the forests, so no permission was requested for the collection. The experiment conditions were established by the research team, and thus no special permission was requested for the experiment. The experiment did not involve any endangered or protected species, and no specific permissions are required for this location to collect plants.

### The species

*Populus deltoides* × *Populus simonii*, a perennial clonal tree, is hybrid poplar with the traits of fast growing, narrow crown, easy rooting and high resistance to abiotic stresses, i.e. flooding and heavy metals stress^42^,^43^,^44^. It is an important economical tree used for high-yield plantation and agro-forestry management in plain regions of eastern and central China^43^,^44^. Due to the advantages of easy rooting from male parent *P. deltoides*, it have high rootage rate of cutting with shoot fragments. Simultaneously, as the capability of root-suckering from female parent *P. simonii*, its roots can extend horizontally and new ramets are produced on these roots. The horizontal roots can generate small fibrous roots^42^. Therefore, *P. deltoides* × *P. simonii* possesses a high ability of sprouting and root suckering. And after deforestation or forest disturbances, the capability of fragment propagations in *P. deltoides* × *P. simonii* may play a significant role in natural re-establishment or afforestation. Shoot and root fragments of *P. deltoides* × *P. simonii* used in this experiment were collected from a four-year *P. deltoides* × *P. simonii* experimental forest (30°28′49″N; 114°21′21″E) of Huazhong Agricultural University, Hubei province, central China. The fragments were from more than 10 clones of *P. deltoides* × *P. simonii* to ensure that the plants collected belong to different genotypes.

### Experimental design

On early April 2014, 400 shoot fragments and 400 root fragments of 0.5 cm (obtained from the apex of the shoot or root) and 2.0 cm in diameter (obtained from the base of the shoot or root) were cut off from the selected clones of the four-year experimental forest population. Each shoot fragment or root fragment contained a centered node, the proximal internode (connected to its adjacent, older part) and the distal internode (connected to its adjacent, younger part). Shoot fragment had only one bud on the node and without other buds on the fragments. Both proximal and distal internodes were at least 7.5 cm long. Of the 800 fragments, 240 shoot fragments and 240 root fragments were selected and used for the experiments, and 40 shoot fragments and 40 root fragments for initial measurements.

The study consisted of two experiments. The first experiment used a four-way factorial randomized block design with 12 replications. The factors were fragment type with two levels (shoot or root), fragment diameter with two levels (0.5 or 2.0 cm), fragment length with two levels (5 or 15 cm) and burial orientation with three levels (horizontal, upward and downward). In this experiment, the fragment internode consisted of both proximal and distal internodes, each having half of the total length. For instance, for the 5 cm length treatment, both the distal and the proximal internode of the fragment were cut into 2.5 cm long. The second experiment employed a three-way factorial randomized block design with 12 replications. There were two levels of fragment type (shoot or root), two treatments of fragment diameter (0.5 or 2.0 cm), combined with four nutrient supply treatments (without nutrient (CK) vs. low frequency vs. high frequency vs. patchy). In this experiment, length of shoot and root both were 15 cm, and burial orientation was horizontal in order to evaluate the effects of nutrient supply treatments.

For the burial treatments, half volume of each fragment was buried under soil in the two experiments. In the upward or downward treatments, either 2.5 or 7.5 cm of distal or proximal aboveground’ part were attached, for a total of 5 or 15 cm of attached part per fragment. In the horizontal treatment, half volume of distal and proximal aboveground’ part were attached. And the bud on the node of shoot was also attached.

Each shoot or root fragment was planted into a plastic pot (diameter = 20 cm, height = 20 cm) filled with 3.5 L of yellow-brown soil (total N = 0.85 g/kg, total P = 0.43 g/kg and total K = 19.52 g/kg) from Shizishan Mountain of our campus. The soil was well mixed before use. In the first experiment, 288 plastic pots were used, 144 for shoot fragments and 144 for root fragments. There were totally 24 treatments. In the second experiment, 192 plastic pots were used for the 96 shoot fragments and the 96 root fragments. In the nutrient treatments, each pot was fertilized with a 0.1% solution of a Peters Professional water-soluble fertilizer (20% N, 20% P_2_O_5_, 20% K_2_O, w⁄w; Scotts, Geldermalsen, Netherlands). We supplied nutrient twice in high frequency and patchy treatments and once in low frequency treatment per week. No nutrient was supplied in CK treatment. The volume of nutrient supply for high frequency, low frequency and CK supplies were 400 mL (200 mL each time), 200 mL and 0 mL per pot, respectively. In the patchiness treatment, each pot was divided into two equal halves (patches), with physical barrier (length = 20 cm, height = 15 cm) between them to prevent the soil water and nutrient in different patches from interacting with each other. Thus, 100 mL of nutrient was applied to the distal shoot and root fragments (i.e. relative young part of the fragment) twice a week, with the total volume of nutrient supply for patchiness treatment being the same as that for low frequency treatment (200 mL per pot). There were totally 16 treatments in the second experiment.

Each pot was irrigated with 200 mL of water every 2–4 days, depending on how fast the soil dried. In patchy treatment, water was added slowly to the soil to avoid massive water flow between adjacent patches. Soil water content was monitored with Soil Moisture Meter TZS-II (HEB Biotech, Xi’an, China) in each treatment during the experimental period. During the experiments, the pots were randomly positioned and reshuffled every week to avoid effects of possible environment differences. All fragments were transplanted on 3 April, 2014. Ten days later, when the clonal fragments started to root, we started the nutrient supply treatments.

The experiments started on 13th April 2014 and ended 15 weeks later on 25th July. All treatments were conducted in the greenhouse experiment bases at Huazhong Agricultural University. During the experiments, the mean temperature was 22.5 °C, measured with Amprobe TR300 (Everett, WA, USA). At the start of the experiments, the average dry weights of the 0.5 cm in diameter & 5 cm in length, 0.5 cm in diameter & 15 cm in length, 2.0 cm in diameter & 5 cm in length and 2.0 cm in diameter & 5 cm in length shoot (root) fragments were 1.613 ± 0.294 (0.871 ± 0.349), 3.898 ± 0.961 (1.308 ± 0.486), 4.443 ± 1.136 (2.748 ± 1.069) and 13.338 ± 3.539 g (8.671 ± 2.642 g) (mean ± SD, n = 10), respectively.

### Measurements and analyses

Before harvest, we first calculated the percentage of survival and the means of size of the survived fragments in each pot. All plants were separated into leaves, shoots (the new emerged shoot) and roots, followed by counting the number of leaves and roots. The old shoot or root fragments were not used in biomass analyses. All parts were then dried at 80 ^o^C for 72 h and weighed. Biomass increment (biomass of the emerged) was the sum of leaves, shoots and roots.

For the first experiment, four-way ANOVA was used to investigate the effects of fragment type (shoot vs. root), fragment diameter (0.5 vs. 2.0 cm), fragment length (5 vs. 15 cm) and burial orientation (horizontal vs. upward vs. downward) on survival, biomass increment, shoot mass, root mass, number of roots and number of leaves of *P. deltoides* × *P. simonii*. For the second experiment, three-way ANOVA was conducted to test the effects of fragment type (shoot vs. root), fragment diameter (0.5 vs. 2.0 cm) and nutrient supply (CK vs. low frequency vs. high frequency vs. patchy) on the measures above mentioned. If significant effects were detected, then *Tukey* multiple comparison tests were used to compare the means between the treatments. All statistical analyses were carried out with SPSS 13.0 (SPSS, Chicago, IL, USA). Prior to ANOVAs, all data were checked for normality and homoscedasticity. The differences were considered to be significant if *P* < 0.05.

## Additional Information

**How to cite this article**: Zhang, P. *et al.* Effects of fragment traits, burial orientation and nutrient supply on survival and growth in *Populus deltoides* × *P. simonii*. *Sci. Rep.* **6**, 21031; doi: 10.1038/srep21031 (2016).

## Figures and Tables

**Table 1:** ANOVAs for effects of fragment type (shoot vs. root), fragment diameter (0.5 vs. 2.0 cm), fragment length (5 vs. 15 cm) and burial orientation (horizontal vs. upward vs. downward) and their interactions on survival and growth measures of final size.

	DF	Survival	Biomass increment	Shoot mass	Root mass	Number of roots	Number of leaves
Fragment type (T)	1	34.35***	8.64**	10.33***	6.75**	18.88***	0.17
Fragment diameter (D)	1	0.07	6.41*	4.14*	9.07**	0.50	0.02
Fragment length (L)	1	70.71***	122.05***	128.98***	98.90***	77.56***	73.33***
Burial orientation (O)	2	2.40	1.08	0.66	1.27	3.14	3.25
T × D	1	1.62	0.03	0.12	0.14	0.01	0.20
T × L	1	0.58	0.68	0.88	2.71	3.99*	6.13*
T × O	2	27.34***	48.38***	47.79***	45.49***	44.87***	30.38***
D × L	1	0.07	5.31*	3.23	7.45**	0.22	0.03
D × O	2	1.23	4.77**	6.01**	1.17	0.67	2.30
L × O	2	1.36	4.52*	3.35*	2.31	2.61	8.90***
T × D × L	1	1.62	0.01	0.01	0.01	0.12	0.17
T × D × O	2	0.46	2.86	2.19	3.97*	0.53	0.53
T × L × O	2	7.60***	35.06***	33.56***	39.54***	28.35***	20.29***
D × L × O	2	1.23	4.82**	6.35**	1.25	0.76	2.09
T × D × L × O	2	0.46	2.42	1.85	3.24	0.73	0.65

Degree of freedom (DF), *F* values and the significance levels (***P < 0.001, **P < 0.01, *P < 0.05) are given.

**Table 2:** ANOVAs for effects of fragment type (shoot vs. root), fragment diameter (0.5 vs. 2.0 cm) and nutrient supply (CK vs. low frequency vs. high frequency vs. patchy) and their interactions on survival and growth measures of final size.

	DF	Survival	Biomass increment	Shoot mass	Root mass	Number of roots	Number of leaves
Fragment type (T)	1	94.97***	65.50***	79.50***	80.73***	92.28***	47.35***
Fragment diameter (D)	1	0.67	5.70*	7.33**	9.61**	16.88***	9.76**
Nutrient supply (N)	3	0.44	1.38	3.33*	2.85*	1.31	0.22
T × D	1	0.01	4.12*	5.68*	7.96**	13.36***	3.95*
T × N	3	1.11	1.10	2.89*	2.76*	1.88	0.19
D × N	3	0.22	0.86	2.12	1.25	0.93	1.04
T × D × N	3	0.44	0.85	1.89	1.30	1.04	0.29

Degree of freedom (DF), *F* values and the significance levels (***P < 0.001, **P < 0.01, *P < 0.05) are given.
